# Activation of angiotensin-converting enzyme 2 ameliorates metabolic syndrome-induced renal damage in rats by renal TLR4 and nuclear transcription factor κB downregulation

**DOI:** 10.3389/fmed.2022.904756

**Published:** 2022-08-12

**Authors:** Heba F. El-Domiaty, Eman Sweed, Mona A. Kora, Nader G. Zaki, Suzan A. Khodir

**Affiliations:** ^1^Department of Medical Physiology, Faculty of Medicine, Menoufia University, Menoufia, Egypt; ^2^Department of Clinical Pharmacology, Faculty of Medicine, Menoufia University, Menoufia, Egypt; ^3^Department of Pathology, Faculty of Medicine, Menoufia University, Menoufia, Egypt; ^4^Department of Anatomy and Embryology, Faculty of Medicine, Menoufia University, Menoufia, Egypt

**Keywords:** metabolic syndrome, diminazene, Ang II, TLR4, NF-κB

## Abstract

**Background:**

Metabolic syndrome (MetS) is an independent risk factor for chronic kidney disease (CKD) through many mechanisms, including activation of the renin–angiotensin system. The deleterious effects of angiotensin II (Ang II) can be counterbalanced by angiotensin-converting enzyme 2 (ACE2). Diminazene aceturate (DIZE), an anti-trypanosomal drug, can activate ACE2.

**Objective:**

This study aimed to investigate the possible reno-protective effects of DIZE in MetS rats with elucidation of related mechanisms.

**Materials and methods:**

Thirty adult male Wistar albino rats were divided equally into control, MetS, and MetS + DIZE groups. Body weight, systolic blood pressure (SBP), and urinary albumin levels were measured. Serum levels of fasting blood glucose (FBG), insulin, uric acid, lipid profile, urea, and creatinine were measured. Homeostasis Model Assessment Index (HOMA-IR) was estimated. Subsequently, renal levels of ACE2, Ang II, malondialdehyde (MDA), reduced glutathione (GSH), and tumor necrosis factor-α (TNF-α) were measured with histopathological and immunohistochemical assessment of TLR4 and NF-κB in renal tissues.

**Results:**

MetS caused dyslipidemia with significant increases in body weight, SBP, FBG, serum insulin, HOMA-IR, uric acid, urea, creatinine, urinary albumin, and renal levels of Ang II, MDA, and TNF-α, whereas renal ACE2 and GSH were significantly decreased. Renal TLR4 and NF-κB immunoreactivity in MetS rats was upregulated. DIZE supplementation of MetS rats induced significant improvements in renal function parameters; this could be explained by the ability of DIZE to activate renal ACE2 and decrease renal Ang II levels with downregulation of renal TLR4 and NF-κB expression.

**Conclusion:**

DIZE exerts a reno-protective effect in MetS, mainly by downregulating renal TLR4 and NF-κB levels.

## Introduction

Metabolic syndrome (MetS) is a cluster of metabolic disorders, including insulin resistance, abdominal obesity, hypertension, dyslipidemia, and glucose intolerance (1). The prevalence of MetS has been increasing over recent periods and has emerged as a major health problem. This may be attributed to modifications to lifestyle and nutritional habits (2). Being a vital metabolic organ, the kidney can be affected by metabolic abnormalities, and there is an increasing prevalence of chronic kidney disease (CKD) among people with MetS (3). A previous experimental study has demonstrated that MetS is associated with renal pathology, including renal hypertrophy, microvascular damage, cortical vasoconstriction, and glomerular hypertension (4). MetS-related renal damage may be clinically manifested by microalbuminuria, abnormal renal tubular function, and increased renal vascular resistance (3). However, the pathophysiological mechanisms of MetS-related renal damage have not been completely elucidated to date.

Inflammation plays a pivotal role in the pathogenesis of MetS. The high cellular fat and sugar levels in MetS enhance the production of pro-inflammatory cytokines, which in turn produce reactive oxygen species (ROS) (5). Pattern recognition receptors such as toll-like receptors (TLRs), which are located in the cell membrane, can detect abnormal molecular patterns that endanger cells (3). TLRs, in particular TLR4, increase cytokine production and mediate innate immune responses through the nuclear transcription factor kappa B (NF-κB) signaling pathway (5). TLR4 has been found to be activated by hyperlipidemia and is upregulated in MetS (6).

It is well known that the renin–angiotensin system (RAS) is found in many tissues and controls various functions. Its overactivation can develop and progress to many pathological conditions such as renal dysfunction and inflammation (7). This can occur through angiotensin-converting enzyme 1 (ACE1), which converts angiotensin I (Ang I) to angiotensin II (Ang II). Ang II exerts its harmful effects by acting on Ang II type 1 receptors (AT1R) (8). Therefore, blocking RAS is used to enhance renal patient clinical outcomes. However, renal diseases continually progress, which increases the need for other therapeutic strategies (9).

On the other hand, angiotensin-converting enzyme 2 (ACE2) antagonizes the adverse effects of Ang II by converting Ang II to Ang 1–7. Ang 1–7 have vasoprotective effects by acting on Mas receptors (10). The balance between harmful ACE1/Ang II and protective ACE2/Ang 1–7 is essential with respect to tissue damage (8).

Diminazene aceturate (DIZE), an anti-trypanosomal drug, was found to activate ACE2 and exerts many protective effects (11). A previous study has revealed the reno-protective effects of DIZE in rats with renal diseases and in diabetic rats (7). However, there are no studies that have reported investigation of the effects of DIZE on MetS-related renal pathology. Moreover, the mechanisms by which DIZE produces reno-protective effects are not completely obvious. Therefore, this study aimed to investigate the possible protective effects of DIZE (ACE2 activator) on MetS-induced renal damage, with exploration of related mechanisms involved.

## Materials and methods

### Sample size calculation and statistical analysis

Sample size was calculated according to the study design and objectives of the study. This study was an experimental one that aimed to investigate the ameliorative effects of ACE2 activator on MetS-induced renal damage in rats, guided by the previous literature (12), and with a CI of 95% and study power of 80% (0.8), the sample size was 30.

### Animals

Thirty adult male Wistar albino rats (150–200 g) of a local strain were used in this study. The animals were left to acclimatize to the environment for 1 week with free access to tap water and routine rodent diet. The study was approved by the Ethics Committee for Scientific Research at the Faculty of Medicine, Menoufia University, Egypt with IRB No. 3/2022PATH7. Animals were treated in accordance with the Guide for the Care and Use of Laboratory Animals (8th edition, The National Academies Press) (13) as well as specific national laws (e.g., the current version of the German Law on the Protection of Animals).

### Experimental design

After acclimatization, rats were randomly divided into three groups (10 rats per group). Each group of rats was placed in two separate cages (5 animals per cage) and labeled as follows:

1- Control group: rats received routine rodent diet.
2- Fructose-induced MetS (MetS) group: rats were fed a 60% high-fructose diet (Technogene Company, Giza, Egypt) plus standard rodent diet for 8 weeks (4, 12). Rats were given distilled water (DIZE vehicle) subcutaneously (s.c.).
3- Fructose-induced MetS DIZE-treated (MetS + DIZE) group: rats that were fed a 60% high-fructose diet plus standard rodent chow were given DIZE (Sigma Aldrich, Steinheim, Germany), dissolved in distilled water at a dose of 15 mg/kg/day, s.c. for 8 weeks, commencing from the first day of high-fructose diet administration (14).

After the end of the experimental period, body weight was measured for all rats, and systolic blood pressure (SBP) was measured using the rat-tail technique. Urine was collected over 24 h periods using metabolic cages for estimation of urinary albumin levels. Then, retro-orbital blood samples were collected after overnight fasting (15). The collected blood samples were left to clot for 10 min and then centrifuged at 4,000 rpm for a further 10 min to isolate serum and kept at -20^°^C for subsequent measurement of fasting blood glucose (FBG), insulin, lipid profile, uric acid, urea, and creatinine serum levels. Homeostasis Model Assessment Index (HOMA-IR) was also estimated. Finally, rats were sacrificed by cervical decapitation, and kidneys were excised. One kidney from each rat was homogenized, and the supernatant was used for measurement of renal levels of ACE2, Ang II, malondialdehyde (MDA), reduced glutathione (GSH), and tumor necrosis factor-α (TNF-α); the other kidney was prepared for histological examination by routine hematoxylin and eosin (H&E) staining and immunohistochemical (IHC) assessment of TLR4 and NF-κB.

### Experimental procedures

#### Measurement of systolic blood pressure

SBP was measured in rats using rat-tail pressure detecting equipment (Harvard Apparatus Ltd., Aden Berge, United Kingdom) connected to a pneumatic transducer (Harvard Apparatus Ltd., Aden Berge, United Kingdom). Changes in pressure were recorded *via* a physiograph (MK III-S; Narco BioSystems Inc., TX, United States).

#### Biochemical assays

##### Measurement of urinary albumin

Urinary microalbuminuria was measured using the immunoturbidimetric method (Gcell Company, Beijing, China).

##### Measurement of fasting blood glucose

FBG was assessed using the enzymatic glucose oxidase method (Biodiagnostic Co., Giza, Egypt) according to the kit instructions.

##### Measurement of serum insulin

Serum insulin concentration was measured using an enzyme-linked immunosorbent assay (ELISA) kit (DRG Instruments GmbH, Marburg, Germany) according to the manufacturer’s instructions.

##### Estimation of homeostasis model assessment index

The insulin resistance index was calculated by HOMA-IR: fasting insulin (MU/mL) × fasting glucose (mg/dL)/405 (16).

##### Measurement of serum uric acid

Colorimetric determination was performed using test reagent kits (Biodiagnostic Co., Giza, Egypt).

##### Measurement of lipid profile

Total cholesterol (TC), triglycerides (TG), low-density lipoprotein-cholesterol (LDL-C), and high-density lipoprotein-cholesterol (HDL-C) concentrations were determined using colorimetric methods (Biodiagnostic Co., Giza, Egypt).

##### Measurement of serum urea and creatinine

Colorimetric kits (Catalog No. 80340; Crystal Chem, Illinois, United States and Biodiagnostic Co., Giza, Egypt) were used to measure serum creatinine and serum urea levels, respectively.

#### Renal homogenate preparation

Kidney specimens were weighed and homogenized separately using a tissue homogenizer (MPW120; MPW Medical Instruments, China). For estimation of renal ACE2 Ang II and TNF-α levels, renal tissues were homogenized in 50 mM phosphate-buffered saline (PBS), pH 7.4. For estimation of renal MDA and renal GSH, renal tissues were homogenized in 10 mM potassium phosphate buffer, pH 7.4. The crude tissue homogenate was centrifuged at 10,000 rpm for 15 min in an ice-cold centrifuge, and the resultant supernatant was collected and stored at -80^°^C for subsequent assays.

#### Measurement of renal angiotensin-converting enzyme 2

Renal ACE2 levels were measured according to ELISA kit instructions (Catalog No. MBS764117; MyBioSource, San Diego, CA, United States).

##### Measurement of renal angiotensin II

Renal Ang II levels were measured according to ELISA kit instructions (Catalog No. E-EL-R1430; Elabscience, Houston, TX, United States).

##### Renal malondialdehyde and glutathione assessment

Renal MDA and GSH were measured using specific colorimetric kits (Biodiagnostic Co., Giza, Egypt) according to the kit instructions.

##### Measurement of renal tumor necrosis factor-α

Renal TNF-α abundance was determined using an ELISA kit (Catalog No. MBS2507393; MyBioSource, San Diego, CA, United States).

#### Histopathological analysis

The other kidney was removed from each rat and fixed in 10% phosphate-buffered formalin solution. The kidneys were stored in 10% neutral buffered formalin and then embedded in paraffin for preparing 4-micron-thick sections. They were stained with H&E for routine histological examination.

##### Immunohistochemical study

**Renal immunohistochemical staining for TLR4 and nuclear transcription factor kappa B.** Several sections were cut from the paraffin-embedded blocks with subsequent steps of deparaffinization and rehydration in xylene and a graded series of alcohol. Antigen retrieval was performed by boiling in 10 mL citrate buffer (pH 6.0) for 20 min, followed by cooling to room temperature. The slides were incubated overnight at room temperature with purified rabbit polyclonal anti-TLR4 antibody (Catalog No. GB11186; Servicebio, Wuhan, Hubei, China) and rabbit monoclonal anti-NF-κB p65 antibody (Catalog No. ab32536, Abcam, Cambridge, United Kingdom). The optimal dilution was 1:1,000 and 1:2,000 for TLR4 and NF-κB, respectively, using PBS. Slides were de-paraffinized using xylene and then rehydrated in decreasing concentrations of ethanol. Antigen retrieval using microwave heating (20 min; 10 mmol/citrate buffer, pH 6.0) after inhibition of endogenous peroxidase activity (hydrogen peroxidase for 15 min) was used. The primary antibody was applied to the slides and incubated overnight at room temperature in a humidified chamber. Sections were washed with PBS and then incubated with secondary antibody for 15 min, followed by further washing with PBS. Finally, detection of bound antibody was accomplished using a modified avidin–biotin labeled reagent followed by 20 min washing with PBS. A 0.1% solution of diaminobenzidine was used for 5 min as a chromogen. Slides were counterstained with Mayer’s hematoxylin for 5–10 min. Rat kidney tissue specimens were used as positive controls. Omission of the primary antibody served as a negative control.

Interpretation of TLR4 and NF-κB IHC results: Regarding TLR4 IHC, brown cytoplasmic, membranous, or membrano-cytoplasmic staining involving any number of cells was considered positive in the studied cases and control specimens (17). Regarding NF-κB IHC, brown cytoplasmic, nuclear, or nucleocytoplasmic staining in any number of cells was considered positive in the studied cases and control specimens (18). Renal tissues in the three studied groups (control, MetS, and MetS + DIZE) were assessed for:

1. **Expression percentage:** Positive cells were counted and given as a percentage of 200 cells of the whole section at 200 × magnification in renal tissues (19).
2. **Intensity of staining:** Graded as mild (+), moderate (++), or strong (+++).
3. **Histo-score (H score):** H score was calculated in all positive specimens according to the following equation: H score = 1 × % of mildly stained cells + 2 × % moderately stained cells + 3 × % of strongly stained cells (20).

#### Statistical analysis

All data were analyzed using SPSS v.16 software (SPSS Inc., Chicago, IL, United States). Shapiro–Wilk tests were performed on all data sets to ensure normal distribution. Results are expressed as mean ± standard deviation (*SD*). The significance of differences between groups was determined by one-way analysis of variance (ANOVA) followed by Tukey’s *post-hoc* test. A *P*-value < 0.05 was considered statistically significant.

## Results

**FIGURE 1:**
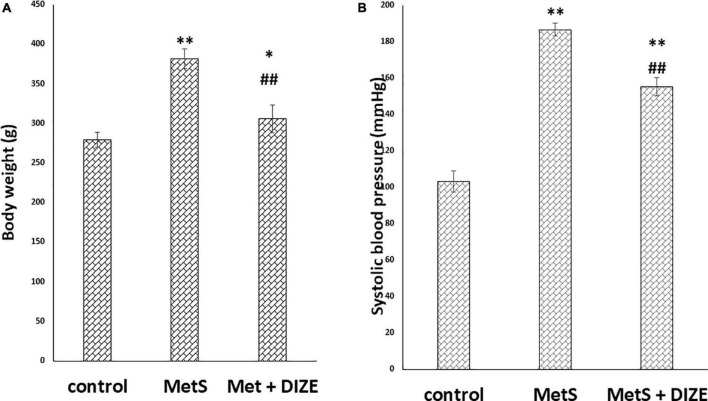
DIZE Effect on **(A)** body weight (g) and **(B)** systolic blood pressure (mmHg) in rats of control, MetS, and MetS + DIZE groups. MetS: fructose-induced metabolic syndrome, MetS + DIZE: fructose-induced metabolic syndrome, DIZE treated. Data are expressed as mean ± *SD* (*n* = 10). ANOVA was used to make group comparisons. *P* < 0.05 considered significant *P < 0.05 significant when compared to control group, ^**^P < 0.001 vs. control group, ^##^*P* < 0.001 vs. MetS group.

The mean value of body weights in the MetS group was significantly higher than those in control group animals (381.9 ± 12.5 g vs. 279.2 ± 9.7 g, respectively, *P* < 0.001), whereas it was significantly lower in the MetS + DIZE group (305.8 ± 17.9 g, *P* < 0.001) than in MetS group animals. Moreover, the mean value of body weights was significantly higher in MetS + DIZE rats than in the control group (*P* < 0.05) (Figure 1A).

The mean values of SBP were significantly higher in the MetS group than in the control group (186.8 ± 3.6 vs. 103.3 ± 5.9 mmHg, respectively, *P* < 0.001). Moreover, the mean values of SBP were significantly lower in MetS + DIZE group rats than in the MetS group (155.2 ± 5 mmHg, *P* < 0.001), but were significantly higher than the control group (*P* < 0.001) (Figure 1B).

### Biochemical results

**FIGURE 2:**
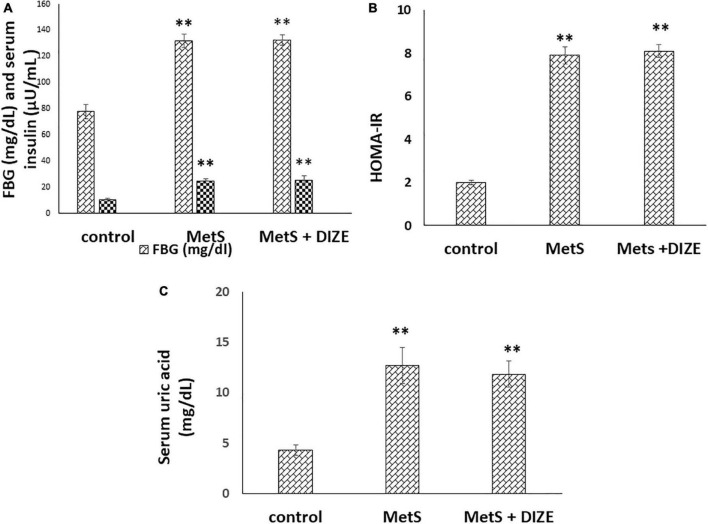
DIZE Effect on **(A)** FBG (mg/dL) and serum insulin (μU/mL), **(B)** HOMA-IR index, and **(C)** serum uric acid (mg/dL) in rats of control, MetS, and MetS + DIZE groups. MetS, fructose-induced metabolic syndrome; MetS + DIZE, fructose-induced metabolic syndrome; DIZE treated. Data are expressed as mean ± *SD* (*n* = 10). ANOVA was used to make group comparisons. *P* < 0.05 considered significant ^**^P < 0.001 vs. control group.

The mean values of FBG were significantly higher in the MetS and MetS + DIZE groups than in control group animals (132 ± 5 and 132.4 ± 4.1 vs. 78 ± 5.5 mg/dL, respectively, *P* < 0.001). However, there was no statistically significant difference in FBG levels between the MetS + DIZE and MetS groups (*P* < 0.05). Moreover, the mean values of serum insulin were significantly higher in MetS and MetS + DIZE group rats than in control group animals (25.1 ± 1.7 and 25.7 ± 3 vs. 10.5 ± 1.3 μU/mL respectively, *P* < 0.001). However, there was no statistically significant difference in serum insulin levels between MetS + DIZE and MetS groups (*P* < 0.05) (Figure 2A).

The mean values of HOMA-IR were significantly higher in MetS and MetS + DIZE groups than in the control group (7.9 ± 0.4 and 8.1 ± 0.3 vs. 2 ± 0.1, respectively, *P* < 0.001). However, there was no statistically significant difference in HOMA-IR between MetS + DIZE and MetS groups (*P* < 0.05) (Figure 2B).

The mean values of serum uric acid were significantly higher in MetS and MetS + DIZE groups than in the control group (12.7 ± 1.8 and 11.8 ± 1.3 vs. 4.3 ± 0.5 mg/dL, respectively, *P* < 0.001). However, there was no statistically significant difference in serum uric acid levels between MetS + DIZE and MetS groups (*P* < 0.05) (Figure 2C).

**FIGURE 3:**
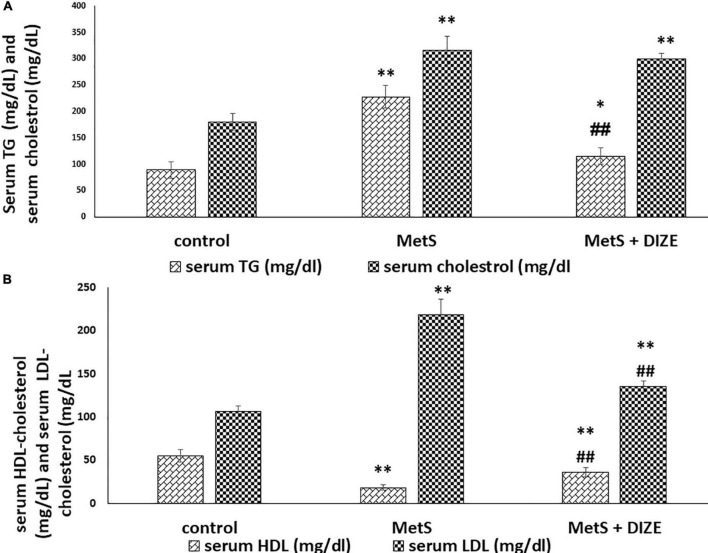
DIZE Effect on lipid profile in rats of control, MetS, and MetS + DIZE groups. **(A)** Serum TG (mg/dL) and serum cholesterol (mg/dL) and **(B)** serum HDL-cholesterol (mg/dL) and serum LDL-cholesterol (mg/dL). MetS, fructose-induced metabolic syndrome; MetS + DIZE, fructose-induced metabolic syndrome; DIZE treated. Data are expressed as mean ± *SD* (*n* = 10). *P* < 0.05 considered significant *P < 0.05 significant when compared to control group, ^**^P < 0.001 vs. control group, ^##^*P* < 0.001 vs. MetS group.

Regarding lipid profiles, the mean values of TC were significantly higher in MetS and MetS + DIZE groups than in the control group (316.3 ± 26.6 and 300.3 ± 10 vs. 179.8 ± 16.9 mg/dL, respectively, *P* < 0.001). However, there was no statistically significant difference in TC levels between MetS + DIZE and MetS groups (*P* < 0.05). Moreover, the mean values of serum TG were significantly higher in MetS group rats than in control group animals (227.9 ± 21.4 vs. 89.4 ± 15.6 mg/dL, respectively, *P* < 0.001). The mean values of serum TG in the MetS + DIZE group were significantly lower than those in the MetS group (115.5 ± 16.1 mg/dL, *P* < 0.001) and significantly higher than those in the control group (*P* < 0.05) (Figure 3A).

The mean values of serum HDL-C were significantly lower in MetS group animals than in control group rats (18.2 ± 2.9 vs. 55 ± 7.4 mg/dL, respectively, *P* < 0.001). HDL-C values were significantly higher in the MetS + DIZE group than in the MetS group (36 ± 5.3 mg/dL, *P* < 0.001) and significantly lower in MetS + DIZE rats than in the control group (*P* < 0.001). Additionally, the mean values of serum LDL-C were significantly higher in MetS group rats than in control group animals (218.4 ± 18.6 vs. 106.5 ± 6.7 mg/dL, respectively, *P* < 0.001). Moreover, it was significantly lower in the MetS + DIZE group than in the MetS group (135.3 ± 6.2 mg/dL, *P* < 0.001) and significantly higher in MetS + DIZE than in the control group (*P* < 0.001) (Figure 3B).

**FIGURE 4:**
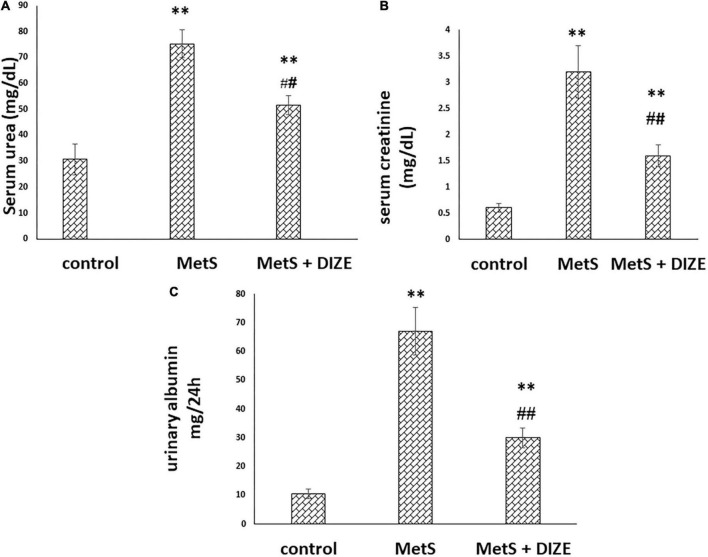
DIZE improves altered renal function in Fructose-induced MetS **(A)** serum urea (mg/dL), **(B)** serum creatinine (mg/dL), and **(C)** urinary albumin (mg/24 h) in rats of control, MetS, and MetS + DIZE groups. MetS, fructose-induced metabolic syndrome; MetS + DIZE, fructose-induced metabolic syndrome; DIZE treated. Data are expressed as mean ± *SD* (*n* = 10). ANOVA was used to make group comparisons *P* < 0.05 considered significant ^**^P < 0.001 vs. control group, ^##^*P* < 0.001 vs. MetS group.

The mean values of serum urea were significantly higher in the MetS group than in the control group (75.3 ± 5.3 vs. 30.7 ± 5.9 mg/dL, respectively, *P* < 0.001). These values were significantly lower in MetS + DIZE group rodents than in MetS group rats (51.6 ± 3.6 mg/dL, *P* < 0.001). However, it was significantly higher in MetS + DIZE animals than in the control group (*P* < 0.001) (Figure 4A).

The mean values of serum creatinine were significantly higher in the MetS group than in the control group (3.2 ± 0.5 vs. 0.6 ± 0.08 mg/dL, respectively, *P* < 0.001). Such values were significantly lower in MetS + DIZE group rats than in MetS group animals (1.6 ± 0.2 mg/dL, *P* < 0.001). However, it was significantly higher in MetS + DIZE rodents than in control group animals (*P* < 0.001) (Figure 4B).

The mean values of urinary albumin were significantly higher in the MetS group than in the control group (67 ± 8.2 vs. 10.6 ± 1.6 mg/24 h, respectively, *P* < 0.001). In contrast, these were significantly lower in the MetS + DIZE group than in the MetS group (30 ± 3.4 mg/24 h, *P* < 0.001). However, it was significantly higher in MetS + DIZE animals than in the control group (*P* < 0.001) (Figure 4C).

**FIGURE 5:**
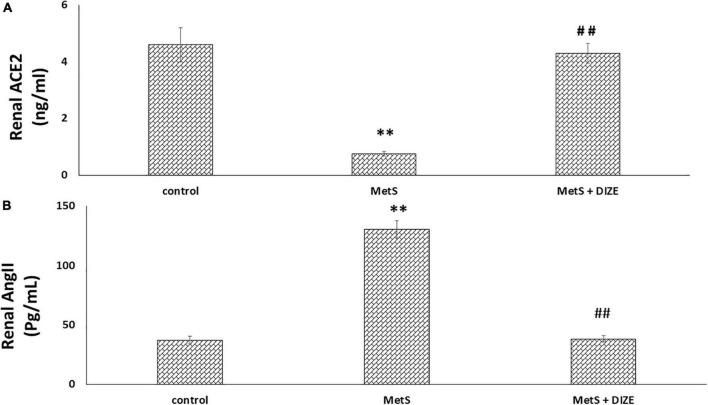
DIZE effect on **(A)** renal ACE2 (ng/ml) and **(B)** renal Ang II (pg/mL) in rats of control, MetS, and MetS + DIZE groups. MetS, fructose-induced metabolic syndrome; MetS + DIZE, fructose-induced metabolic syndrome; DIZE treated. Data are expressed as mean ± *SD* (*n* = 10). ANOVA was used to make group comparisons. *P* < 0.05 considered significant ^**^P < 0.001 vs. control group, ^##^*P* < 0.001 vs. MetS group.

The mean values of renal ACE2 were significantly lower in the MetS group than in the control group (0.75 ± 0.08 vs. 4.6 ± 0.62 ng/mL, respectively, *P* < 0.001), but were significantly higher in MetS + DIZE group rats than in MetS group animals (4.3 ± 0.35 ng/mL, *P* < 0.001). However, there was no statistically significant difference in renal ACE2 levels between MetS + DIZE and control group animals (*P* < 0.05) (Figure 5A).

The mean values of renal Ang II were significantly higher in the MetS group than in the control group (130.5 ± 7.3 vs. 37.4 ± 3.1 pg/mL, respectively, *P* < 0.001), but were significantly lower in MetS + DIZE group rats than in MetS group animals (38.3 ± 2.7 pg/mL, *P* < 0.001). However, there was no statistically significant difference in renal Ang II levels between MetS + DIZE and control group animals (*P* < 0.05) (Figure 5B).

**FIGURE 6:**
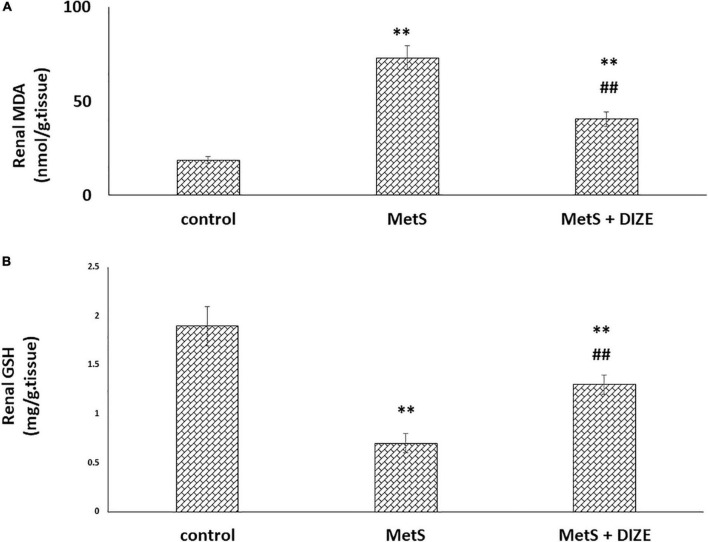
Antioxidative stress effect of DIZE in Fructose-induced MetS **(A)** renal MDA (nmol/g tissue) and **(B)** GSH (mg/g tissue) in rats of control, MetS, and MetS + DIZE groups MetS, fructose-induced metabolic syndrome, MetS + DIZE, fructose-induced metabolic syndrome; DIZE treated. Data are expressed as mean ± *SD* (*n* = 10). ANOVA was used to make group comparisons. *P* < 0.05 considered significant ^**^P < 0.001 vs. control group, ^##^*P* < 0.001 vs. MetS group.

The mean values of renal MDA were significantly higher in MetS group animals than in the control group (73.1 ± 6.3 vs. 18.6 ± 1.9 nmol/g tissue, respectively, *P* < 0.001), whereas they were significantly lower in the MetS + DIZE group than in the MetS group (40.4 ± 3.9 nmol/g tissue, *P* < 0.001). Moreover, these values were significantly higher in MetS + DIZE rodents than in control group rats (*P* < 0.001) (Figure 6A).

The mean values of renal GSH levels were significantly lower in the MetS group than in the control group (0.7 ± 0.1 vs. 1.9 ± 0.2 mg/g tissue, respectively, *P* < 0.001), whereas these were significantly higher in MetS + DIZE group rats than in MetS group animals (1.3 ± 0.1 mg/g tissue, *P* < 0.001). Moreover, they were significantly lower in MetS + DIZE rats than in the control group (*P* < 0.001) (Figure 6B).

**FIGURE 7:**
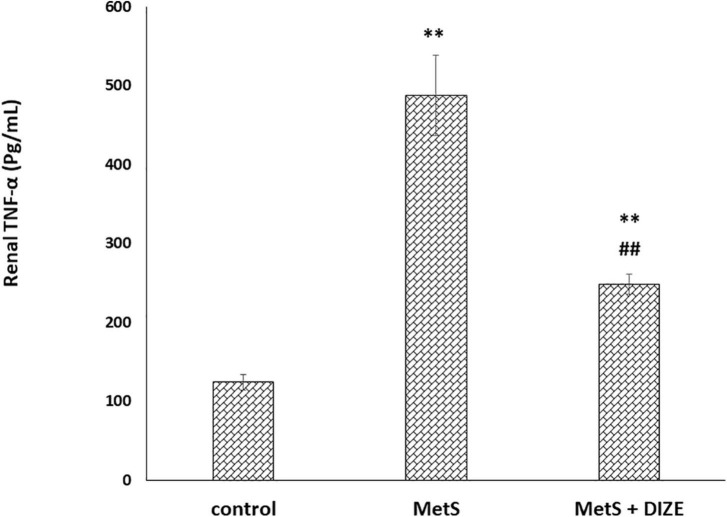
DIZE effect on serum TNF-α (pg/mL) in rats of control, MetS, and MetS + DIZE groups. MetS, fructose-induced metabolic syndrome; MetS + DIZE, fructose-induced metabolic syndrome; DIZE treated. Data are expressed as mean ± *SD* (*n* = 10). ANOVA was used to make group comparisons. *P* < 0.05 considered significant ^**^P < 0.001 vs. control group, ^##^*P* < 0.001 vs. MetS group.

The mean values of renal TNF-α were significantly higher in the MetS group than in the control group (487.1 ± 50.8 vs. 124.3 ± 9.9 pg/mL, respectively, *P* < 0.001), whereas they were significantly lower in MetS + DIZE group animals than in MetS group rats (248 ± 12.9 pg/mL, *P* < 0.001). Moreover, they were significantly higher in the MetS + DIZE group than in the control group (*P* < 0.001) (Figure 7).

### Histopathological results

**FIGURE 8:**
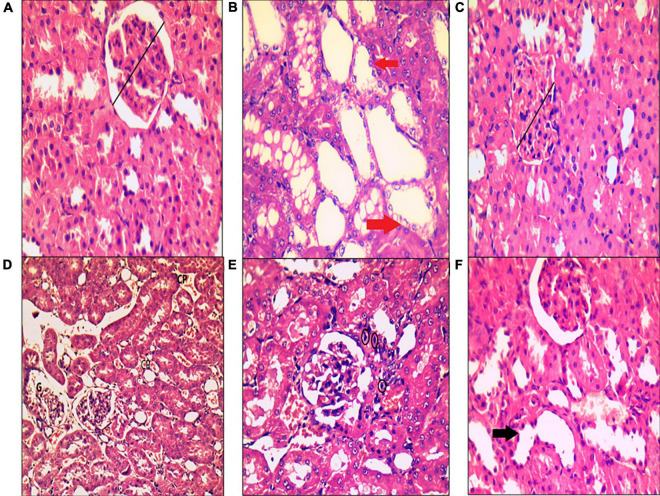
Histopathological examination of kidney tissue after hematoxylin and eosin staining in rats of the control group **(A)**, MetS group **(B–E)**, and MetS + DIZE group **(F)**. The control group **(A)** exhibited no morphological changes, with preserved glomerular width (black lines). The MetS + DIZE group **(F)** showed more or less normal morphology other than some dilated tubules (black arrow). The MetS group **(B–E)** showed degeneration of tubular epithelial cells with tubular dilatation (red arrows), decreased width of glomerulus compared to the control group (black lines), and peritubular dilated and congested blood capillaries (CP) with congested dilated glomerular capillaries (G). Lymphocytic inflammatory infiltrate (black circles) was also observed. (Magnification: 400 × for **A–C,E,F** and 200 × for **D**).

In the control group, H&E staining revealed normal architecture in the cortex and medulla, with normal appearance of the renal corpuscles and tubules. Most cells of the proximal, distal convoluted, and collecting tubules were normal (Figure 8A). In the MetS group, deterioration of renal histology in the form of degeneration of tubular epithelial cells with tubular dilatation (Figure 8B), decreased width of glomerulus compared to the control group (Figure 8C), peritubular dilated and congested blood capillaries together with congested dilated glomerular capillaries (Figure 8D), and lymphocytic inflammatory infiltrate (Figure 8E) were observed. The MetS + DIZE group exhibited a picture rather like control group kidneys except for a small number of dilated tubules (Figure 8F).

#### TLR4 immunohistochemical

**FIGURE 9:**
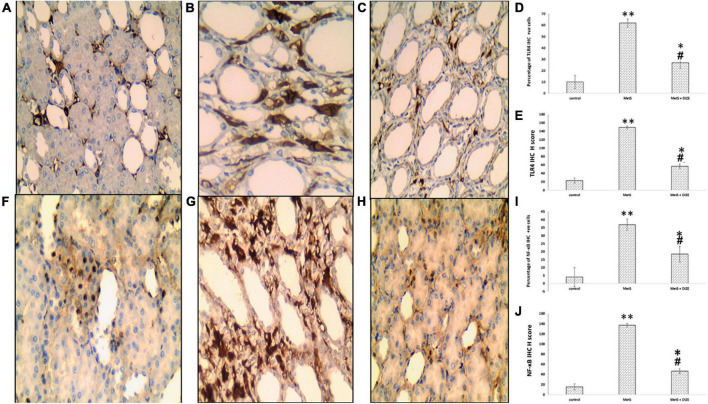
TLR4 and NF-κB IHC staining of control, MetS, and MetS + DIZE groups. TLR4: strong membrano-cytoplasmic staining in both tubular and glomerular cells (the inset at lower right corner clarifies glomerular area) in the MetS group **(B)** compared to both control (red arrow points to glomerular cells and black arrow points to tubular cells) **(A)** and MetS + DIZE groups **(C)**. NF-κB: strong nucleocytoplasmic staining in both tubular and glomerular cells (the inset at lower left corner clarifies glomerular area) in the MetS group **(G)** compared to moderate nuclear staining in both control **(F)** and MetS + DIZE groups (red arrow points to glomerular cells and black arrow points to tubular cells) **(H)** (IHC 400 × magnification for all). Effect of DIZE on TLR4 and NF-κB percentage and H score among control, MetS, and MetS + DIZE groups. MetS, fructose-induced metabolic syndrome; MetS + DIZE, fructose-induced metabolic syndrome; DIZE treated. Data are expressed as mean ± *SD* (*n* = 10). *P < 0.05 vs. control group, ^#^*P* < 0.05 vs. MetS. ^**^P < 0.001 vs. control group, ^##^*P* < 0.001 vs. MetS **(D,E,I,J)**.

Immunostaining for TLR4 in the control, MetS, and MetS + DIZE groups revealed moderate to strong membrano-cytoplasmic staining in both glomeruli and tubules (Figures 9A–C). The mean value of the percentage of positive cells in the MetS group was significantly higher than that in the control group (62.00 ± 20.97 vs. 10 ± 0.00, respectively, *P* < 0.001). After supplementation with DIZE, the mean value of the percentage of positive cells was significantly lower in MetS + DIZE rats (27.0 ± 11.59, *P* < 0.05) than in the MetS group, but was still significantly higher in MetS + DIZE animals than in the control group (*P* < 0.05) (Figure 9D).

Regarding TLR4 H scores, the mean value of TLR4 H scores was significantly higher in MetS group rats than in the control group (149.0 ± 38.42 vs. 23.18 ± 6.03, respectively, *P* < 0.001). Moreover, the mean values of TLR4 H scores were significantly lower in the MetS + DIZE group than in the MetS group (57.0 ± 18.28, *P* < 0.05), but significantly higher than the control group (*P* < 0.05) (Figure 9E).

#### Nuclear transcription factor kappa B immunohistochemical

Immunostaining for NF-κB in control, MetS, and MetS + DIZE group rats revealed moderate to strong nucleocytoplasmic staining in both glomeruli and tubules (Figures 9F–H). The mean value of the percentage of positive cells was significantly higher in the MetS group than in the control group (37.0 ± 17.02 vs. 4.09 ± 1.04 respectively, *P* < 0.001). The mean percentage of positive cells was significantly lower in MetS + DIZE group rats than in MetS group (18.5 ± 6.68, *P* < 0.001) and significantly higher than the control group (*P* < 0.05) (Figure 9I).

Regarding NF-κB H scores, the mean values of NF-κB H scores were significantly higher in the MetS group than in the control group (137.0 ± 27.1 vs. 15.45 ± 4.16 respectively, *P* < 0.001), whereas they were significantly lower in the MetS + DIZE group than in the MetS group (46.5 ± 21.35, *P* < 0.05). Moreover, the values were significantly higher in MetS + DIZE animals than in the control group (*P* < 0.05) (Figure 9J).

## Discussion

MetS is an independent risk factor for CKD, because the prevalence of MetS is higher in patients with CKD than in those with non-CKD. On the other hand, there is a higher prevalence of CKD among populations with MetS (21). MetS can induce renal parenchymal damage and microvascular remodeling (22, 23). Many pathological mechanisms have been identified to induce renal dysfunction in MetS, such as RAS activation, dyslipidemia chronic inflammation, and oxidative stress (21). However, the pathways involving MetS-induced renal damage are still unclear.

Although blocking of RAS either by ACE1 or by angiotensin-receptor blockers is of good prognostic value in patients with CKD (24), another alternative arm of RAS may result in better effects. The other arm of RAS involves ACE2/Ang 1–7, which counterbalances the undesired effects of Ang II. Many studies have demonstrated that ACE2 is more important than ACE in creating a state of balance in RAS. The deficiency in ACE2, whether it is congenital or acquired affected markedly the tissue level of Ang II. While ACE deficiency did not significantly affect Ang II tissue level (25, 26). ACE2 is reported to be activated by DIZE, an anti-trypanosomal drug, which mediates a beneficial effect in various pathological conditions. Therefore, this study aimed to identify the possible reno-protective effects of DIZE (ACE2 activator) in a rat model of MetS, with exploration of the underlying mechanisms.

In the current study, a 60% high-fructose diet for 8 weeks was effective in producing the clinical manifestations of MetS. This diet produced a significant increase in rat body weights, SBP, FBG, and serum uric acid levels, as well as dyslipidemia and insulin resistance. Furthermore, there was a significant increase in the serum levels of urea, creatinine, and urinary albumin levels, which identifies the renal damaging effects of MetS. The present study demonstrated that DIZE administration to MetS rats significantly decreased body weight and SBP and improved dyslipidemia. Moreover, DIZE ameliorated renal function parameters. For exploration of the possible reno-protective mechanisms involving DIZE, renal levels of ACE2, Ang II, MDA, GSH, and TNF-α levels were measured, in addition to the expression of TLR4 and NF-kB detected by IHC in the rat kidney.

Fructose is the most widely used sugar in the food industry, employed as a sweetener, and is naturally found in fruits and vegetables (27). It was selected in this study to mediate an animal model of MetS, as the consumption of fructose has greatly increased over recent years, which is consistent with the high incidence of obesity and MetS (28). The high hepatic influx of fructose has a lipogenic effect, which impairs the insulin signaling pathway causing insulin resistance and dyslipidemia (29). Previously, it has been proven that a high-fructose diet increases plasma non-esterified fatty acid levels, which represents evidence of insulin resistance (27, 30). The hyperuricemic effect of a high-fructose diet can be explained by the unique ability of fructose to increase uric acid production (31). Moreover, uric acid has been identified to mediate oxidative stress state, stimulate local RAS, and increase the expression of pro-inflammatory chemokines, which induces renal injury (32).

Additionally, the renal damaging effect of fructose-induced MetS was parallel to the study results of Mansour et al. (31) and Yildirim (33), in which there were significant increase in serum urea and creatinine levels. This is in accordance with our histopathological findings, which revealed that a high-fructose diet induced kidney remodeling, resulting in kidney injury and deterioration of renal histology. There was degeneration of tubular epithelial cells with tubular dilatation, peritubular dilated and congested blood capillaries, together with congested dilated glomerular capillaries, and lymphocytic inflammatory infiltrate, which were compatible with other previous studies (2, 34).

The damaging effect of MetS on the rat kidney could be attributed to RAS activation and ROS production, which were identified in this study by elevated renal Ang II, and MDA levels and decreased renal ACE2 and GSH level. Many studies have demonstrated an association between high plasma aldosterone levels and insulin resistance with MetS (35–37). Additionally, it has been found that obesity, hyperglycemia, and hypertension induce RAS activation (38). Ang II, by acting on AT1R, plays a role in ROS production and salt-sensitive hypertension by stimulating sodium reabsorption (39). Furthermore, Ang II induces efferent arteriolar vasoconstriction, which causes glomerular hypertension and hyperfiltration (40). The resulting glomerular hyperfiltration causes albuminuria, which is consistent with the results of this study. Additionally, albuminuria may cause chronic tubulointerstitial injury *via* podocyte damage (41).

Intracellular ROS mediated by Ang II activate many intracellular mediators that induce inflammation and tissue injury (42). The kidney is an important target organ for inflammation, and renal inflammatory reactions mediate further ROS production, renal fibrosis, and glomerulosclerosis (21). In this study, the elevated renal expression of TNF-α, as a pro-inflammatory marker together with upregulated TLR4 IHC expression in renal tissues of MetS rats, was consistent with a study by Hardy et al. who stated that MetS elevates the expression of *TNF*-α, *TLR4*, *TLR2*, and *IL6* genes in peripheral monocytes of adolescents (6). Similarly, Nair et al. detected increased gene and protein expression of TLR4 with suppressed antioxidant function, and this was associated with renal dysfunction in obese Zucker fatty rats (5). Moreover, the current study revealed a significant elevation in renal NF-κB immunostaining in MetS rats compared to controls. TLR4 is an innate immune receptor that detects pathogens endangering the cell and mediates pro-inflammatory cytokine production in different tissues (43). The TLR4 pathway triggers a downstream signaling cascade, which activates the transcription factor NF-κB, thereby controlling pro-inflammatory cytokine production. These results suggested that TLR4, *via* its downstream effector molecule NF-κB, plays a key role in the renal inflammatory effects and renal damage mediated by Ang II in MetS.

DIZE is proven to have a reno-protective effect in a rat model of ischemia/reperfusion injury (44), and in subtotal nephrectomy, DIZE decreases renal cortical ACE expression and elevates renal ACE2 expression (8). However, the reno-protective effect of DIZE in MetS-induced renal damage has not been studied previously. Our results demonstrated that supplementation of MetS rats with DIZE significantly improved renal function parameters, and this finding is parallel to the renal histopathological results, which revealed that DIZE attenuated renal tubular and glomerular damage, suggesting that DIZE has a reno-protective effect against MetS-induced renal injury. This is consistent with the results of previous studies, which have demonstrated the reno-protective effects of DIZE in different rat models such as gentamicin-induced acute renal injury (45) and γ-irradiation induced renal damage (46). This ameliorative effect can be explained by the significant reduction in renal Ang II levels in MetS rats supplemented with DIZE, compared to MetS rats. Here, the reduced renal Ang II was secondary to significantly increased renal ACE2. It is known that ACE2 antagonizes the deleterious effects of ACE/Ang II by degrading Ang II to Ang 1–7. DIZE, by acting as ACE2 activator, can improve many pathological conditions mediated by Ang II, such as inflammation, vasoconstriction, fibrosis, and oxidative stress (8); this explains the improved renal oxidative stress observed in MetS rats supplemented with DIZE.

Furthermore, DIZE was found to have a renal anti-inflammatory effect in this study, as it caused a significant reduction in renal TNF-α levels compared to MetS rats. This finding is supported by the anti-inflammatory effect of DIZE, as described by Chen et al. against acute myocardial infarction in rats (47). The renal anti-inflammatory effect of DIZE can be explained by the current histopathological findings. The expression of TLR4 and NF-κB detected by IHC was significantly decreased in rat kidney tissue after DIZE supplementation, compared to the MetS group. This result can be supported by Kuriakose et al. who stated that DIZE mediates its anti-inflammatory properties by reducing NF-κB expression, as detected by western blotting (48). Therefore, the renal anti-inflammatory effect of DIZE can be attributed to suppression of the renal TLR4–NF-κB pathway. The ability of DIZE to suppress the renal TLR4–NF-κB pathway was related to ACE2 activation, which caused hydrolysis of Ang II and hence antagonizing the renal inflammatory effect of Ang II. Ang 1–7, the products of Ang II hydrolysis by ACE2, mediate its protective effects mainly by acting on Mas receptors (49).

The current study demonstrated other beneficial effects of DIZE with respect to body weight, lipid profile, and SBP. These results are in accord with those of Stachowicz et al. (50), who proved that DIZE elevates plasma HDL-C levels and decreases hepatic TG levels in female apolipoprotein E-knockout mice fed a high-fat diet (HFD). Additionally, Singh et al. found that Ang 1–7 significantly decrease serum TG levels and increase serum HDL-C abundance with no significant effects on serum TC levels in diabetic rats (51). Furthermore, Bruce et al. detected that HFD rats treated with DIZE show significant reduction in food intake, white adipose tissue, and body weight (14). The antihypertensive effect of DIZE was previously detected in several studies (52–54), which can be explained by the vasodilatory effects of DIZE *via* activation of the ACE2/Ang-(1–7)/Mas axis with increased nitric oxide release, as demonstrated by the study of De Maria et al. (11).

## Conclusion

DIZE, by acting as an ACE2 activator, exerts a reno-protective effect in MetS rats through downregulation of the renal TLR4–NF-κB pathway, thereby mediating renal anti-inflammatory and antioxidant properties. Hence, DIZE may be considered a promising future therapy for protection against MetS-induced renal damage.

## Data availability statement

The original contributions presented in this study are included in the article/supplementary material, further inquiries can be directed to the corresponding author/s.

## Ethics statement

The animal study was reviewed and approved by the Ethics Committee for Scientific Research at the Faculty of Medicine, Menoufia University, Egypt with IRB No. 3/2022PATH7.

## Author contributions

All authors contributed to the concept and design of the work and contributed to drafting, revising the manuscript, final approval, and agreement of the final version.
